# Firing the Sting: Chemically Induced Discharge of Cnidae Reveals Novel Proteins and Peptides from Box Jellyfish (*Chironex fleckeri*) Venom

**DOI:** 10.3390/toxins7030936

**Published:** 2015-03-18

**Authors:** Mahdokht Jouiaei, Nicholas R. Casewell, Angel A. Yanagihara, Amanda Nouwens, Bronwen W. Cribb, Darryl Whitehead, Timothy N. W. Jackson, Syed A. Ali, Simon C. Wagstaff, Ivan Koludarov, Paul Alewood, Jay Hansen, Bryan G. Fry

**Affiliations:** 1 Venom Evolution Lab, School of Biological Sciences, the University of Queensland, St. Lucia, QLD 4072, Australia; E-Mails: m.jouiaei@uq.edu.au (M.J.); tnwjackson@gmail.com (T.N.W.J.); dr.syedabidali@gmail.com (S.A.A.); jcoludar@gmail.com (I.K.); mjhansen@uqconnect.edu.au (J.H.); 2 Institute for Molecular Bioscience, the University of Queensland, St. Lucia, QLD 4072, Australia; E-Mail: p.alewood@imb.uq.edu.au; 3 Alistair Reid Venom Research Unit, Liverpool School of Tropical Medicine, Liverpool L3 5QA, UK; E-Mail: nicholas.casewell@lstmed.ac.uk; 4 Pacific Cnidaria Research Lab, Department of Tropical Medicine, University of Hawaii, Honolulu, HI 96822, USA; E-Mail: ayanagih@hawaii.edu; 5 School of Chemistry and Molecular Biosciences, the University of Queensland, St. Lucia, QLD 4072, Australia; E-Mail: a.nouwens@uq.edu.au; 6 Centre for Microscopy & Microanalysis and School of Biological Sciences, the University of Queensland, St. Lucia, QLD 4072, Australia; E-Mail: b.cribb@uq.edu.au; 7 School of Biomedical Sciences, the University of Queensland, St. Lucia, QLD 4072, Australia; E-Mail: darryl.whitehead@uq.edu.au; 8 HEJ Research Institute of Chemistry, International Centre for Chemical and Biological Sciences (ICCBS), University of Karachi, Karachi-75270, Pakistan; 9 Bioinformatics Unit, Liverpool School of Tropical Medicine, Liverpool L3 5QA, UK; E-Mail: Simon.Wagstaff@lstmed.ac.uk

**Keywords:** *Chironex fleckeri*, transcriptome, proteome, nematocyst, pressure induced disruption, ethanol induced discharge

## Abstract

Cnidarian venom research has lagged behind other toxinological fields due to technical difficulties in recovery of the complex venom from the microscopic nematocysts. Here we report a newly developed rapid, repeatable and cost effective technique of venom preparation, using ethanol to induce nematocyst discharge and to recover venom contents in one step. Our model species was the Australian box jellyfish (*Chironex fleckeri*), which has a notable impact on public health. By utilizing scanning electron microscopy and light microscopy, we examined nematocyst external morphology before and after ethanol treatment and verified nematocyst discharge. Further, to investigate nematocyst content or “venom” recovery, we utilized both top-down and bottom-up transcriptomics–proteomics approaches and compared the proteome profile of this new ethanol recovery based method to a previously reported high activity and recovery protocol, based upon density purified intact cnidae and pressure induced disruption. In addition to recovering previously characterized box jellyfish toxins, including CfTX-A/B and CfTX-1, we recovered putative metalloproteases and novel expression of a small serine protease inhibitor. This study not only reveals a much more complex toxin profile of Australian box jellyfish venom but also suggests that ethanol extraction method could augment future cnidarian venom proteomics research efforts.

## 1. Introduction

Stinging cells (cnidocytes) are distinctive of venomous marine animals of *Cnidaria* phylum. They contain microscopic organelles (cnidae) that discharge explosively, injecting a mixture of compounds into prey or potential predators [1,2]. Upon contact with human skin or other surface (e.g., prey and predator), penetrant cnidae or nematocysts evert harpoon-like tubules laden with spines that act like hypodermic devices to inject venom (proteinaceous porins, neurotoxic peptides and bioactive lipids) [3,4,5]. Envenomation syndromes induced by cnidarian animals represent a therapeutic challenge especially to bathers, swimmers and surfers. Envenomation symptoms are painful hemorrhagic skin lesions, systemic reactions (e.g., direct effects on muscle and nerve tissue), long-term immunological responses, and occasionally fatalities due to *Chironex fleckeri* cardiovascular and pore-forming toxins [6,7,8,9].

In recent years, cnidarian venoms have begun to be investigated as a potential source of novel bioactive therapeutic compounds [10,11,12,13,14]. However, in comparison with the vast number of studies conducted on the venoms of other venomous animals such as snakes, cone snails, spiders and scorpions, cnidarian venoms have received scant attention from toxinologists. The principal technical impediment in cnidarian research is the fact that the venom does not exist in a large discrete gland as an aqueous mixture in milligram quantities but instead is distributed in microscopic individual nematocysts, each containing picogram of protein [7]. Venom analysis at the picogram scale presents challenges as cnidarian venom is a complex mixture of bioactive molecules, some of which are aqueous while others are lipidic [7,8]. As a consequence, a modern proteomics approach based on high-throughput mass spectrometry analysis is ideal [15].

Previous venom preparation techniques have been based upon electrical discharge of nematocysts through human amnion [16], or homogenization [17] and pulverization or maceration [18] of whole frozen tentacles and saline or phosphate buffer wash. However, the “venom” recovered utilizing these methods is in fact total tentacular extracts comprised of the contents of both nematocysts and other tentacle cell types. Current venom preparation techniques are based on mechanical rupture of the isolated nematocysts with mortar and pestle grinding [19], glass beads [20] and sonication [9,21] in the presence of extraction solutions such as distilled water or saline. However, difficulties relating to equipment availability and contamination of the venom by structural components (e.g., nematocyst capsule-walls) are the major disadvantages of a mechanical disruption and solvent based extraction approach. Another approach has been developed in which density purified intact cnidae are disrupted using pressure followed by rapid centrifugation to harvest the contents without contaminating then with tentacular material or structural components [7]. This technique results in venom with very high specific activity and complexity but is a laborious process.

Since certain chemicals such as ethanol, or 5% acetic acid in distilled water, cause massive cnidae discharge in some cnidarian species [22,23,24] (*Hydrozoa* and *Cubozoa*, respectively), in this study we utilized ethanol to obtain venom proteins and peptides from box jellyfish, *C. fleckeri*, because of its significant envenomation consequences and need of opportune therapeutic tools [7,8]. This study highlights the advantages of this new technique, which results in pure venom, free of contaminants from the tentacles or structural components of nematocysts.

## 2. Results and Discussion

The venom composition of *C. fleckeri* has previously been studied, although the methodological approaches used to obtain venom varied between authors [16,17,18,19,20,21]. Moreover, due to the lack of a transcriptomic database underpinning the annotation of the isolated proteins, proteomic approaches were unlikely to discover novel toxins unique to this species. Notably, when pulverization based approaches are used on purified nematocysts in combination with a solvent extraction, the recovered proteins include structural components of the nematocyst capsule rather than just intra-capsular material. Even more concerning is that many “venom” preparations are in fact solvent extracts of the whole tentacles, and thus contain all tentacular biomolecules soluble in the chosen solvent. In addition, many current venom obtaining techniques based upon mechanical disruption of nematocysts are time-consuming and expensive. Here we report a new venom recovery technique based on chemically induced discharge of nematocysts that maximizes and accelerates the identification of the toxic molecules comprised in jellyfish venom. Also in order to rule out that the identified proteins are produced by tentacular epithelial cells, (e.g., toxin Nv1 localized to ectodermal gland cells in the tentacles rather than nematocysts [25]), we have isolated nematocysts from *C. fleckeri*, disrupted them *in vitro* and analyzed and compared the released protein mixture with identified proteins in our method.

### 2.1. Microscopy Examination of Undischarged and Chemically Discharged Nematocysts

Thus far, there have been differences in the reported nematocyst types and morphology of *C. fleckeri* cnidome. Despite this diversity in the results of studies conducted by various research groups [19,26,27,28], the consensus is that the cnidome includes four types of nematocyst: (i) those that contain the lethal venom component (microbasic *p*-mastigophores); (ii) those that penetrate the prey’s skin or cuticle and ensnare it with hook-like structures in order to secure close contact with the tentacles (small and large tri-rhopaloids); (iii) adherent cnidae which adhere to the prey via a coiled shaft upon discharge (holotrichous isorhizas); and (iv) enigmatic spineless adhesive cnidae that secrete sticky fluid (atrichous isorhizas) [19,26,27,28].

**Figure 1:**
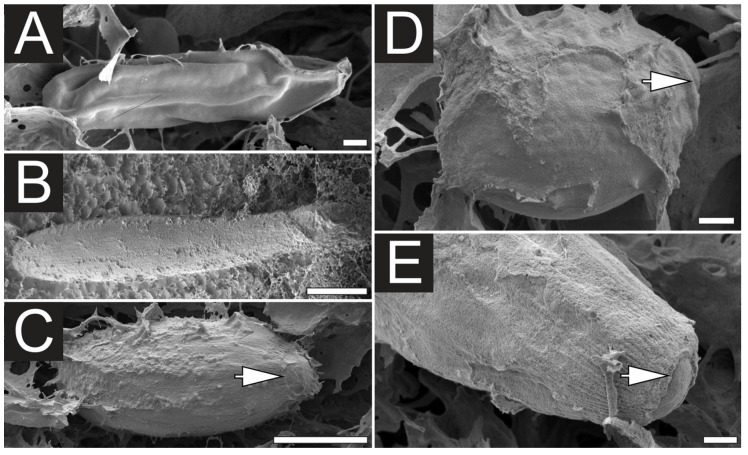
Scanning electron microscopy (SEM) of undischarged *C. fleckeri* nematocysts. (**A**) Undischarged rod-shaped atrichous isorhiza; (**B**) banana-form microbasic *p*-mastigophore; (**C**) Large oval *p*-rhopaloid; (**D**) Small sub-spherical *p*-rhopaloid; (**E**) Detail of operculum (the door of the capsule) of an oval *p*-rhopaloid. The operculum (solid arrows) is found to be a convex shape, which upon discharge, part and permit the tubule and capsule components to be released. Scale bars, **A**, **D**, **E** 1 µm; **B**, **C** 10 µm.

**Figure 2:**
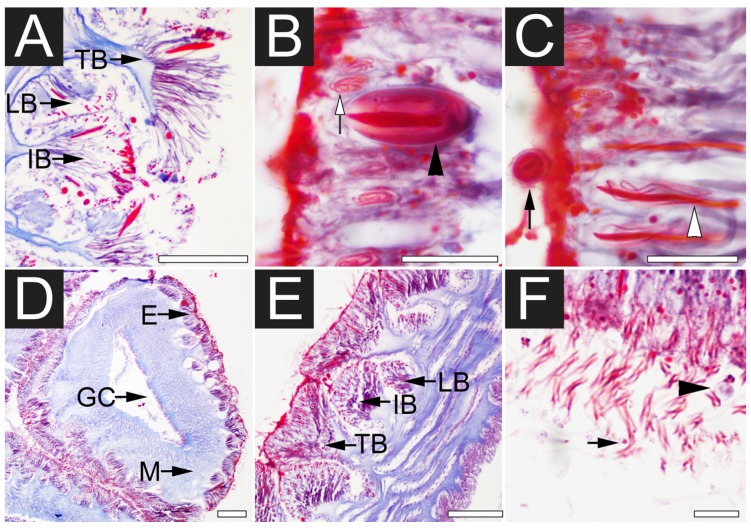
Light microscopy (LM) of *C. fleckeri* nematocysts histochemically stained with Masson’s Trichrome. (**A**) The orientation of undischarged nematocysts in longitudinal section showing top batteries (TB), intermediate batteries (IB) and lower batteries (LB); (**B**) Undischarged large oval *p*-rhopaloid (black arrowhead) and atrichous isorhiza (white arrow); (**C**) Undischarged microbasic *p*-mastigophores (white arrowhead) and small sub-spherical *p*-rhopaloid (black arrow); (**D**) Discharged tentacular axis region showing gastrovascular cavity (GC), mesoglea (M), and epidermis (E); (**E**) Detail of epidermis evagination with ethanol discharged nematocysts. Note the nematocyst batteries; (**F**) Part of the epidermis showing the extruded tubules (black arrow). Note the empty capsule (black arrowhead). Scale bars, **A**, **D**, **E**, **F** 100 µm; **B**, **C** 50 µm.

**Figure 3:**
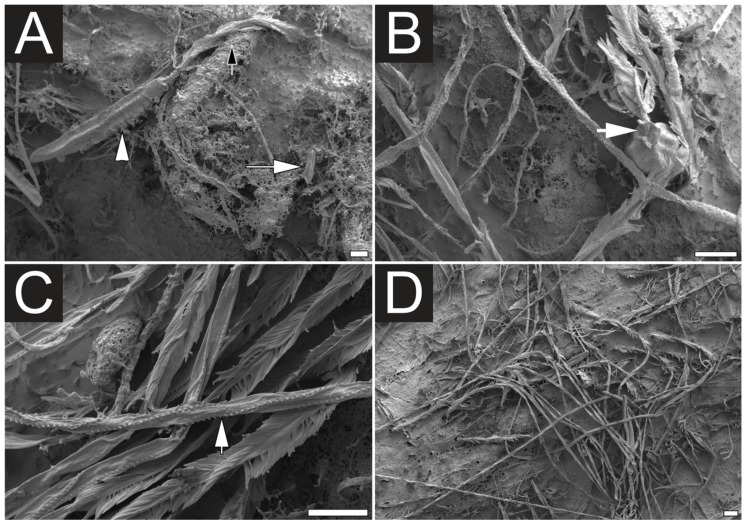
Scanning electron microscopy (SEM) of ethanol discharged *C. fleckeri* nematocysts. (**A**) A discharged microbasic *p*-mastigophore (white arrowhead) with the extrude shaft (black arrow) and an atrichous isorhiza (white arrow); (**B**) Small sub-spherical *p*-rhopaloid. Note the operculum (white arrow); (**C**) High magnification SEM of shafts and tubules of microbasic *p*-mastigophores. Note the spines (white arrow); (**D**) Sublimed surface of a discharged tentacle. Scale bars, 10 µm.

In this study the effectiveness of ethanol in inducing discharge discharge *C. fleckeri* nematocysts was proved by both light microscopy and scanning electron microscopy (SEM). Prior to immersion in ethanol, SEM examination of tentacles revealed undischarged nematocysts, which were categorized as rod-shaped atrichous isorhizas (Figure 1A and Figure 2B), banana-form microbasic *p*-mastigophores (Figure 1B and Figure 2C), large oval *p*-rhopaloids (Figure 1C,E, and Figure 2B) and small sub-spherical *p*-rhopaloids (Figure 1D and Figure 2C). In order to achieve a better understanding of nematocyst orientation within the tissue and the morphological characteristics of the discharged and undischarged nematocysts, the histological samples of tentacles were examined using light microscopy (Figure 2). The transverse section of the tentacle clearly showed three groups of nematocyst batteries: top, intermediate and lower (Figure 2A,E); with nematocysts located at the tips of the batteries. Before chemical discharge, tubules were observed to be coiled and twisted inside the intact nematocyst capsule (Figure 2A–C). After discharge, the capsule remained intact, although the capsular components including shaft, tubule and venom were expelled (Figure 2D–F and Figure 3A–C). Moreover, after immersion in ethanol, the tentacle surface was found to be densely packed with discharged nematocysts (Figure 3D), with a few nematocysts, mostly those placed in lower nematocyst batteries remain undischarged (Figure 2E). As previously suggested [22], the “roofed-over” effect of the top nematocyst batteries likely prevents the less-prominent intermediate and inferior batteries from being exposed to ethanol. It should be mention that this new ethanol recovery based method demonstrated higher yield in term of the number of proteins identified. Therefore, it is reasonable to propose that this method is more effective in obtaining a good yield of venom, as compared to a previously reported high activity and recovery protocol. The effect of ethanol on nematocyst discharge in *C. fleckeri* is in line with what previously reported, showing that ethanol stimulate nematocyst discharge of various species of jellyfish [22,23,24]. On the other hand, our observation differ from the inhibitory effect of ethanol on chemoactivation of *in situ* discharge in *Pelagia noctiluca* (Cnidaria, Scyphozoa) oral arms [29]. This inconsistency is probabely due to specimens difference.

### 2.2. Transcriptome Assembly and Functional Annotation

A total of 2,973,873 reads were obtained for the *C. fleckeri* tentacle transcriptome. Automated assembly resulted in 5128 contigs (Table S1). To classify the putative function of the resulting contigs we annotated them using BLAST2GO—from a total of 5128 contigs, 59.7% of them were successfully identified by BLAST annotation and we subsequently classified these contigs according to biological process, cellular component and molecular function, respectively. A complete description of the contigs, BLAST statistics, contigs classification and top-hit species can be found in Table S1.

### 2.3. Comparative Proteomic Analyses and Identified Proteins

ProteinPilot analysis of the *C. fleckeri* pressure disrupted nematocysts (PDN) proteome retrieved 175 proteins (with 99% high-confidence spectra and a 1% FDR), representing 3.41% of the translated amino acid sequences sourced from the tentacle transcriptome (Table S2). The same approach applied to the chemically discharged nematocysts (CDN) proteome resulted in the identification of 241 proteins (with 99% high-confidence spectra and a 1% FDR), representing 4.69% of the sequences found in the transcriptome (Table S3). Further gene ontology analyses by BLAST2GO software classified the two sets of proteomes into 10, 18 and 13 categories according to their cellular component, biological process, and molecular function, respectively (Figure S1).

On comparing the proteomic data retrieved from PDN and CDN, 129 of 175 PDN proteins were found in the CDN set (73.71%). On the other hand, from 241 of the proteins identified in the CDN proteome, 134 proteins (55.60%) were identified in the PDN proteome. The identified proteins from each of these samples are summarized and displayed in Table S4.

Through our combined proteomic and transcriptomic approach, we identified newly described *C. fleckeri* haemolysin toxins *CfTX-A* (UniProt: T1PRE3) and *CfTX-B* (UniProt: T1PQV6) and potent cardiotoxic toxin *CfTX-1* (UniProt: A7L035) [30] in venom obtained from both PDN and CDN. Interestingly, the *CfTX-2* (UniProt: A7L036) gene identified in our tentacle transcriptome was only identified in the CDN venom sample—where it exhibited high proteomic coverage (42.39%)—and was completely absent from PDN venom sample. This pattern was observed with other protein types, as our chemical discharge approach resulted in a larger number of protein identifications and higher confidence values for the identified proteins (Table 1).

We detected several metalloproteases containing ShK and astacin domains in the proteome of PDN and CDN that have not been previously identified in *C. fleckeri* venom. The ShK-containing metalloproteases have recently been characterized in mammalian proteomes where they display remarkable sequence similarity to BgK and ShK toxins from sea anemones [31]. Although two of the identified metalloproteases containing the ShK domain do not exhibit sequence homology to ShK and BgK toxins from sea anemones, suggesting that they do not possess potassium channel blocker activity. In addition to ShK domain, we found astacin domains (metalloprotease M12A family) in the metalloproteases of the *C. fleckeri* venom. The metalloprotease M12A family has been recruited into the venom systems of numerous animals, including the spider *Loxosceles intermedia* [32], centipedes [33], and the jellyfish *Stomolophus meleagris* [34]. These proteins are thought to be responsible for degrading the extracellular matrix, thereby facilitating the diffusion of other venom components to their molecular targets. Their presence in the nematocysts of the sea anemone *Nematostella vectensis* has been associated with defense rather than prey capture [35]. Although transcripts encoding proteases containing M12A and ShK domains were found in both PDN and CDN venoms here, their evolutionary history and present function in *C. fleckeri* venom remain unclear. However, given the additional experimental evidence of the presence of metalloproteases in cnidarian nematocyst venoms [34,35], it is probable that *C. fleckeri* metalloproteases play a role in prey capture, prey digestion or defense (or a combination thereof).

A plesiotypic serine protease-inhibitor (kazal-type) was found in the CDN sample. Recently, kazal-containing transcripts have been found in the sea cucumber *Holothuria glaberrima* where they are associated with a defensive/immunity role in the inactivation of bacterial proteases [36]. The possible role of this toxin in *C. fleckeri* venom remains unknown.

**Table 1:** Summary of venom proteins/peptides identified from *C. fleckeri* venom material. Pressure disrupted nematocysts (PDN); Chemically discharged nematocysts (CDN); a: comparative protein-level results across multiple searches using ProteinPilot; b: The percentage of matching amino acids from identified peptides having confidence greater than or equal to 95%; c: The number of distinct peptides having at least 95% confidence.

Protein ID Transcriptome Match	Uniprot Accession #(s)/Best Cnidaria BLAST Hit	Conserved Domain	Comparative Protein Level ^a^		%Cov (95%) ^b^		Matched Peptides # (%95) ^c^	
			PDN	CDN	PDN	CDN	PDN	CDN
**Known Cubozoa Toxins**								
**T0179**	T1PQV6/Toxin B precursor	–	44	6	65.11	60.15	8	63
**T0362**	T1PRE3/Toxin A precursor	–	25	7	29.7	55.3	12	49
**T2746**	A7L035/Toxin *CfTX-1* precursor	–	93	40	45.9	77.9	3	14
**T2621**	A7L036/Toxin *CfTX-2* precursor	–	Not found	68	Not found	42.4	Not found	10
**Metalloproteases**								
**T0344**	A7S336; Predicted protein; Nematostella vectensis	ShKT domain (IPR003582)	47	27	16.92	23.69	6	27
**T0690**	A7SNJ4; Metalloendopeptidase; Nematostella vectensis	Peptidase M12A, astacin domain (IPR001506); ShKT domain (IPR003582)	72	61	17.49	45.73	4	16
**T2821**	A7S5S4; Metalloendopeptidase; Nematostella vectensis	Peptidase M12A, astacin domain (IPR001506)	177	77	25	47.5	2	12
**T1091**	–	Peptidase M12A, astacin domain (IPR001506)	207	189	12.7	7.7	1	2
**T2460**	A7T0S0; Metalloendopeptidase; Nematostella vectensis	Peptidase M12A, astacin domain (IPR001506)	138	200	25.6	41	3	7
**Serine Protease inhibitors**								
**T0134**	A7SCV8; Predicted protein; Nematostella vectensis	Kazal domain (IPR002350)	Not found	149	Not found	5.34	Not found	3

## 3. Experimental Section

### 3.1. Specimen Collection

Mature live specimens of *C. fleckeri* were collected by dip net from Weipa, Queensland, Australia during the spring 2013 by Bryan Fry and Nicholas Casewell. For transcriptome analysis, fresh tentacles were dissected out manually then immediately frozen in liquid nitrogen and subsequently stored at −80 °C. For histology and cryo-SEM studies, samples of tissue were dissected from the tips of both untreated and ethanol-treated tentacles, and then fixed in 10% neutral-buffered formalin (NBF) at pH 7.2.

### 3.2. Nematocyst Morphology

#### 3.2.1. Cryo-Scanning Electron Microscopy

Longitudinal and transverse sections (<4 mm long) of the untreated and ethanol-treated tentacles that had previously been fixed in NBF were washed in phosphate buffered saline (PBS) at pH 7.2 for 5 × 10 min then transferred to glutaraldehyde solution (3% in phosphate buffer, pH 7.2) for permanent preservation. Samples were frozen and examined with a Gatan Alto 2500 cryo-system on a JEOL JSM-7100F field emission scanning electron microscope (JEOL Ltd., Tokyo, Japan). Separately, untreated and ethanol-treated sections (3 × 5 × 2 mm: height, length and width) were placed in a metal flange-sample holder, locked into position and immediately frozen by immersing in liquid nitrogen. The samples were transferred into the cryo-system, and the tissue was either fractured with a blade (undischarged samples) or left intact (discharged samples). Subsequently, the water was removed from the surface region by raising the temperature to −80 °C for 20, 15 or 7 min, depending on the desired extent of sublimation of the ice. The sample was then coated with platinum to 5–7 nm and viewed at 2 kV. Images were stored digitally without modification except for adjustment of contrast and brightness. Cryo-fixation rapidly freezes the water component in a sample to form ice through heat exchange with the liquid nitrogen slush, producing a solid sample that can be fractured or viewed whole. The sublimation step avoids damage to the delicate, hydrated tissue caused by chemical drying and allows 3D visualization of structures within that tissue.

#### 3.2.2. Histology

Ultra-structural analysis of the tentacles was carried out using histological sections of intact tentacle tips. Before processing, NBF fixed specimens were washed in PBS (pH 7.2) for 5 × 10 min to remove the fixative. The samples were then dehydrated in an ethanol series (70% × 45 min, 90% × 45 min, 100% × 45 min), followed by paraffin wax embedding (2 × 45 min). Serial transverse sections, 6 µm in thickness, were taken at intervals along the length of the tentacles using a Hyrax M25 Rotary Microtome (Carl Zeiss, Jena, Germany). The sections were then stained with Masson’s Trichrome stain which gives clear distinction between collagen fiber, cytoplasm and nucleus [37]. The slides were then observed by differential interference contrast microscopy (10–100 × magnification).

### 3.3. Transcriptome Library Construction

*Chironex fleckeri* tentacles were preserved in liquid nitrogen prior to use. Total RNA was isolated from 800 mg of frozen tentacle using the standard TRIzol Plus RNA purification kit (Life Technologies, Carlsbad, CA, USA). RNA quality was assessed using a Bioanalyser (Agilent, Santa Clara, CA, USA) and ribosomal RNA removed using the Ribo-Zero rRNA Removal Kit for Human/Mouse/Rat (Epicenter, Madison, WI, USA). The RNA-Seq library was prepared from 50 ng of the enriched RNA material using the ScriptSeq v2 RNA-Seq Library Preparation Kit (epicentre), following 12 cycles of amplification. The sequencing library was purified using AMPure XP beads (Agencourt, Brea, CA, USA), quantified using the Qubit dsDNA HS Assay Kit (Life Technologies) and the size distribution assessed using a Bioanalyser (Agilent). The library was sequenced on a single lane of an Illumina MiSeq machine housed at the Centre for Genomic Research, Liverpool, UK, generating 2,973,873 reads representing 1,481,186 read pairs. The ensuing read data was quality processed, first by removing the presence of any adapter sequences using Cutadapt (https://code.google.com/p/cutadapt/) and then by trimming low quality bases using Sickle (https://github.com/najoshi/sickle). Reads were trimmed if bases at the 3' end matched the adapter sequence for 3 bp or more, and further trimmed with a minimum window quality score of 20. After trimming, reads shorter than 10 bp were removed. Paired-end read data was assembled into 5128 contigs using the de-novo transcriptome assembler VTBuilder [38] executed with the following parameters: min. transcript length 150 bp; min. read length 150 bp; min. isoform similarity 96%. Assembled contigs were annotated with the BLAST2GO Pro v3 [39,40] using the BLASTX algorithm with a significance threshold of 1e-3, to provide BLAST annotations against NCBI’s non redundant (NR) protein database release 67 followed by mapping to gene ontology terms, and Interpro domain annotation using default parameters. Some sequences annotated ambiguously were confirmed using Expasy (UniProt Knowledgebase (Swiss-Prot + TrEMBL), Uniref100) comparison. Additionally, sequences identified proteomically were scanned for the presence of signal peptide with SignalP 4.1 Server [41], and checked for functional domains and gene ontology (GO) with InterPro [42] and the Conserved domain Architecture Retrieval Tool. Contigs were then translated using CLC Genomics Workbench 5 (CLC bio, Aarhus, Denmark) to provide all 6 reading frames to provide a sequence database for the proteomic characterization of the venom components. Trimmed raw sequencing reads have been deposited in the SRA database of NCBI (http://www.ncbi.nlm.nih.gov/sra) with the BioProject identifier PRJNA273442. Assembled contigs can be found in Supplementary File 1 and BLAST2GO annotation files are available by request from the corresponding author.

### 3.4. Venom Extraction

#### 3.4.1. Chemically Induced Discharge of Nematocysts by Ethanol

In order to induce chemical discharge of nematocysts, fresh live tentacles of a mature specimen were immersed in 1 liter absolute ethanol for 30 s at room temperature. The tentacle was then removed and the ethanol transferred to −80 °C to precipitate the proteins. After 24 h of precipitation, the material was centrifuged at 14,000× *g*, 4 °C for 30 min. The supernatant was decanted and the pellets containing protein extracts stored at −80 °C for further use.

#### 3.4.2. Pressure Disrupted, Pre-Purified Nematocysts

Venom was provided by Dr. Angel A. Yanagihara. Specifically undischarged nematocysts were purified and then disrupted en masse using a French Press 20 K pressure cell (SLM-AMINCO Cat# FA078) subjected to a quick 90 s 10,000× *g* spin and snap frozen in liquid nitrogen as previously described [7]. The venom was stored at −80 °C for proteomic analyses.

### 3.5. Proteomics Methods

Sample preparation for the AB SCIEX 5600 mass spectrometer was performed according to the protocol described previously [43]. Briefly, the venom pellets generated by CDN and PDN were dissolved in 8 M urea, 50 mM ammonium bicarbonate buffer. Sample protein concentrations were determined using the 2D Quant Kit (GE Healthcare, Piscataway, NJ, USA). The equivalent of 200 µg of the CDN and PDN venoms were reduced with 5 mM dithiothreitol at 30 °C for 45 min and alkylated with 25 mM idoacetamide for 30 min at RT in the dark. Samples were diluted 1:4 with 50 mM ammonium bicarbonate buffer followed by tryptic digestion (Sigma–Aldrich, St. Louis, MO, USA) at 100:1 protein: trypsin ratio at 37 °C overnight, before they were freeze-dried. Samples were then diluted with 5% acetonitrile (ACN)/0.1% formic acid (FA) and subsequently desalted with a C18 Toptip (Glygen, Columbia, MD, USA) using: (i) 100% ACN to wet the resin (3 × 150 mL); (ii) 5% ACN/0.1% trifluoroacetic acid (3 × 150 mL) for tip equilibration and washing steps; and (iii) 80% ACN/0.1% TFA (2 × 150 mL) for elution. The eluted protein fragments were freeze-dried and then resuspended in 0.5% acetic acid/2% ACN for further analyses.

The proteins fragments were separated by SCX chromatography on an Agilent 1100 chromatography system. 50 µL of each sample were injected onto a Zorbax 300-SCX column (5 µm, 4.6 × 50 mm) (Agilent) at a flow rate of 500 µL/min and gradient of 0–5 min, 0% buffer B; 5–25 min, 0%–50% buffer B; 25–27 min, 50%–80% buffer B; 27–32 min, 80% buffer B; 32–34 min, 80%–0% buffer B. buffer B was held at 80% for 5 min for washing the column and returned to 1% buffer B for equilibration prior to the next sample injection. Buffer A consisted of 0.5% acetic acid/2% ACN and buffer B contained 0.5% acetic acid/2% ACN/250 mM ammonium acetate. A total of 72 fractions (250 µL) were collected and pooled to give 10 fractions. Pooled fractions were de-salted with C18 Toptip (Glygen) and were analyzed by LC-MS/MS on a Shimadzu Prominence Nano HPLC (Shimadzu, Kyoto, Japan) coupled to a Triple TOF 5600 mass spectrometer (ABSciex, Concord, ON, Canada) equipped with a Nanospray III interface. The samples were first de-salted on an Agilent C18 trap (0.3 × 5 mm, 5 mm) for 8 min at 30 µL/min. The trap column was then placed in-line with Vydac Everest C18 (300 A, 5 µm, 150 mm × 150 um) column for mass spectrometry analysis. Linear gradients of 10%–60% solvent B over 45 min at 1 µL/min flow rate was used for peptide elution where buffer A consisted of 1% ACN/0.1% FA and buffer B contained 80% ACN/0.1% FA.

Full scan TOF-MS data was acquired over the mass range 350–1600 and for product ion ms/ms 40–1600. The mass spectrometer acquired 500 ms full scan TOF-MS data followed by 20 by 50 ms full scan product ion high sensitivity mode. A collision energy spread (CE = 40 ± 15 V) was used for fragmentation. The acquired MS data were processed using Analyst TF 1.6.1 software (ABSciex). Proteins were identified by database searching using ProteinPilot v 4.5 (ABSciex) with the Paragon Algorithm using fasta formatted protein sequences for the *C. fleckeri* translated proteins obtained from the assembled tentacle transcriptome and all publicly available *C. fleckeri* venom sequences using the UniProt database. Search parameters were defined as a thorough search using trypsin digestion enzyme iodoacetamide cysteine alkylation emphasis on biological modifications and “thorough” search setting. Peptides were considered identified if they could be established at greater than 99.0% probability, and proteins were considered identified if they could be established at greater than 99.0% probability and contained at least 2 identified peptides and all matched spectra were confirmed by manual inspection. The accepted false discovery rates (FDR) for peptides and proteins were less than or equal to 1% [44].

In order compare the protein level and quantitative results across venom obtained by CDN and PDN, the protein summary of both techniques were submitted to Protein Alignment Template (V2.000p, ABSciex, Canada), where the reference protein ID and aligned protein ID were designated to PDN and CDN, respectively. This yielded a high percent of set proteins being matched with the reference proteins, allowing a fair comparison of the high quality ID from each set.

## 4. Conclusions

Here we have provided the first detailed description of the *C. fleckeri* nematocyst transcriptome and used this data to facilitate accurate identification of venom components detected by proteomic techniques. This combination of approaches permitted robust comparisons of the composition of the venom proteome from both pressure disrupted and chemically discharged nematocysts. Whilst both approaches yielded a variety of proteins, our novel approach using ethanol as a chemical stimulus to trigger discharge of jellyfish nematocysts led to the identification of more venom toxins than the pressure disruption technique. Further studies could determine the relative toxicity in terms of hemolytic units (HU50) recovered per microgram of protein obtain by ethanol discharge in comparison to those recovered by pressure disruption. In summary, we have demonstrated that the use of ethanol as a chemical stimulant for discharge and an extraction solvent for obtaining venom proteins from jellyfish tentacles has great potential for future cnidarian venom research. This method facilitates accurate venom protein identification and provides a simple, cost-effective approach that circumvents the time and technical limitations associated with mechanical disruption.

## Supplementary Materials

Supplementary materials can be accessed at: http://www.mdpi.com/2072-6651/7/3/0936/s1.

## Abbreviation

PDN: Pressure disrupted nematocysts
CDN: chemically discharged nematocysts
LC: liquid chromatography
MS/MS: tandem MS
SPI: serine protease inhibitor
NBF: 10% neutral-buffered formalin
contig: contiguous
PBS: phosphate buffered saline
