# Supplementary material for: Firing the Sting: Chemically Induced Discharge of Cnidae Reveals Novel Proteins and Peptides from Box Jellyfish (Chironex fleckeri) Venom
Source: Toxins (Basel). 2015 Mar 18;7(3):936–50. doi: 10.3390/toxins7030936 (PMC4379534; doi:10.3390/toxins7030936)
Supplement: Supplementary file 1 [file toxins-07-00936-s001.zip › New folder/toxins-76019-Figure S1.pdf]

Supplementary Information

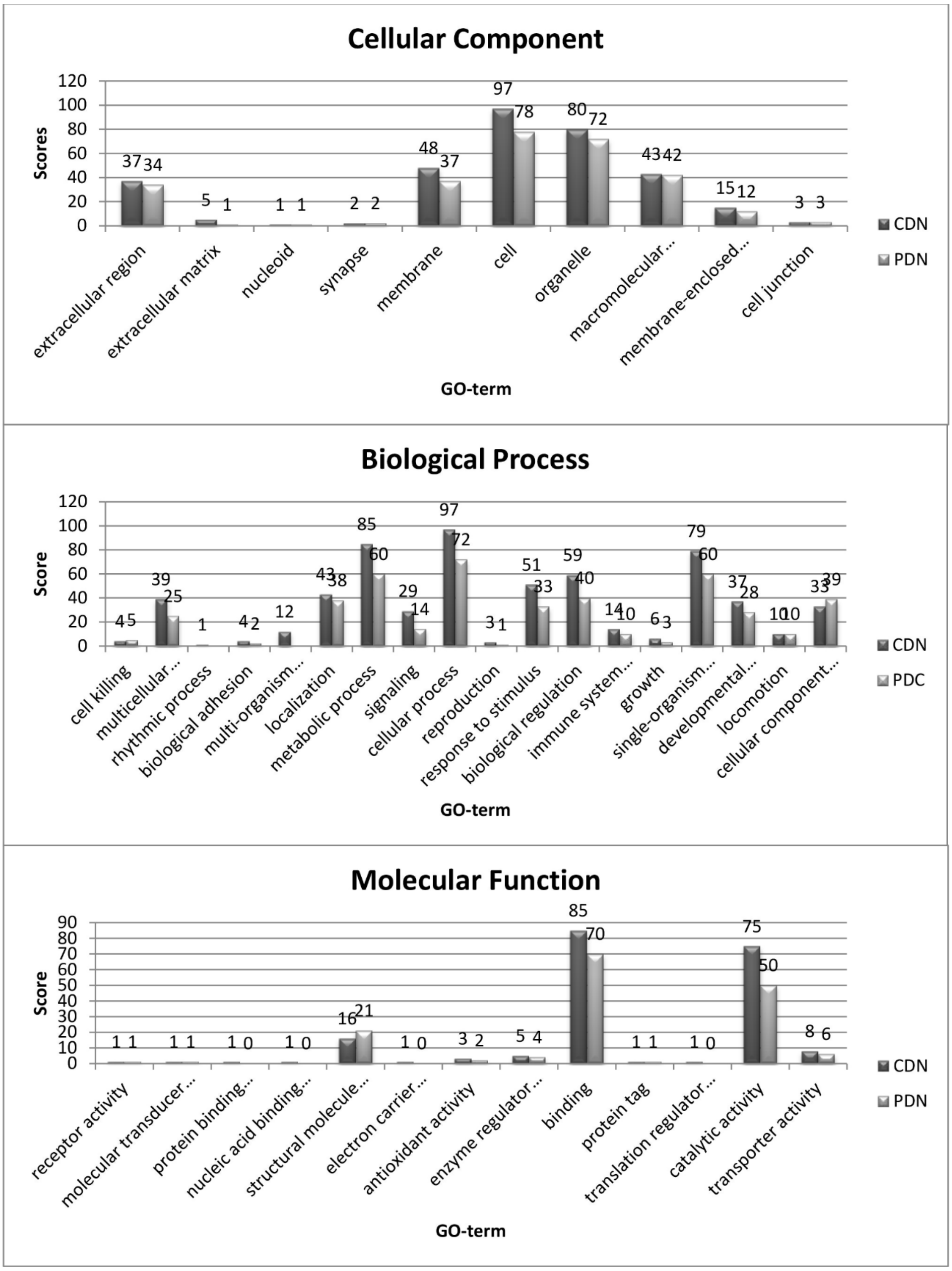

Figure S1. The relative abundance of proteins present in chemically discharged nematocysts (CDN; dark grey bars) and pressure disrupted nematocysts (PDN; light grey bars) based on GO-term classification of the assembled and annotated *C. fleckeri* transcriptome.
